# Implementing competing risks in discrete event simulation: the event-specific probabilities and distributions approach

**DOI:** 10.3389/fphar.2023.1255021

**Published:** 2023-10-30

**Authors:** Fanny Franchini, Victor Fedyashov, Maarten J. IJzerman, Koen Degeling

**Affiliations:** ^1^ Cancer Health Services Research, Centre for Health Policy, Melbourne School of Population and Global Health, Faculty of Medicine, Dentistry and Health Sciences, The University of Melbourne, Melbourne, VIC, Australia; ^2^ Cancer Health Services Research, Centre for Cancer Research, Faculty of Medicine, Dentistry and Health Sciences, The University of Melbourne, Melbourne, VIC, Australia; ^3^ ARC Training Centre in Cognitive Computing for Medical Technologies, The University of Melbourne, Parkville, VIC, Australia; ^4^ Department of Cancer Research, Peter MacCallum Cancer Centre, Melbourne, Australia; ^5^ Erasmus School of Health Policy & Management, Erasmus University, Rotterdam, Netherlands

**Keywords:** discrete event simulation, competing risks modelling, censored data, frequentist implementation, bayesian framework

## Abstract

**Background:** Although several strategies for modelling competing events in discrete event simulation (DES) exist, a methodological gap for the event-specific probabilities and distributions (ESPD) approach when dealing with censored data remains. This study defines and illustrates the ESPD strategy for censored data.

**Methods:** The ESPD approach assumes that events are generated through a two-step process. First, the type of event is selected according to some (unknown) mixture proportions. Next, the times of occurrence of the events are sampled from a corresponding survival distribution. Both of these steps can be modelled based on covariates. Performance was evaluated through a simulation study, considering sample size and levels of censoring. Additionally, an oncology-related case study was conducted to assess the ability to produce realistic results, and to demonstrate its implementation using both frequentist and Bayesian frameworks in R.

**Results:** The simulation study showed good performance of the ESPD approach, with accuracy decreasing as sample sizes decreased and censoring levels increased. The average relative absolute error of the event probability (95%-confidence interval) ranged from 0.04 (0.00; 0.10) to 0.23 (0.01; 0.66) for 60% censoring and sample size 50, showing that increased censoring and decreased sample size resulted in lower accuracy. The approach yielded realistic results in the case study.

**Discussion:** The ESPD approach can be used to model competing events in DES based on censored data. Further research is warranted to compare the approach to other modelling approaches for DES, and to evaluate its usefulness in estimating cumulative event incidences in a broader context.

## 1 Introduction

Discrete event simulation (DES) is increasingly used to model disease, treatment, and care delivery pathways in healthcare (Günal and Pidd, 2017; Vazquez-Serrano et al., 2021). Given its event-based handling of time and the ability to account for resource capacity constraints, it is an effective and efficient individual-level (or microsimulation) modelling technique for a range of decision problems (Marshall et al., 2020). The increased flexibility of DES compared to more traditional approaches, such as state-transition modelling, also implies that certain decisions regarding the model structure and methodologies used may not necessarily be applicable to such traditional approaches and adjustments must be made when implementing such a dynamic model (Karnon et al., 2012).

Competing risks or events are common in healthcare and clinical studies (Pintilie, 2006; Koller et al., 2012; Coemans et al., 2022) and refer to a situation where there are multiple possible outcomes that can occur, and the occurrence of one outcome precludes the occurrence of the others or changes their likelihood. One of the advantages of DES is the ability to implement competing risks using different approaches (Barton et al., 2004; Karnon et al., 2012). In implementing decision-analytic models, every transition in the model pathway typically involves competing events. More specifically, if it is possible to move to more than one model state from a certain state, the transitions to these subsequent states are competing risks. For example, for a model structure commonly used in oncology defined by three health states (i.e., disease free, recurrence, and death), the possible transitions to the recurrence or death state from the disease-free state are competing risks. Similarly, in a model of patient flows in an emergency department, discharging a patient after triaging by a nurse may be a competing event relative to the patient being referred to an emergency doctor for further investigations.

The ability to model competing risks using different strategies allows the modeler to select the approach that best suits the available evidence and context (Caro and Möller, 2014). Each strategy necessitates defining a data analysis framework and the required simulation steps, collectively referred to as a modelling approach. In terms of competing risks, two broad approaches to time-to-event estimation are commonly used. When considering competing risks, there are two broad approaches to time-to-event estimation (Barton et al., 2004). The first calculates individual time estimates for each potential subsequent event and proceeds based on which event is predicted to occur earliest. The second approach also generates an overall time estimate for the next event but employs an additional sampling process to identify the specific type of event likely to happen. Importantly, the likelihood of each event type occurring can be influenced by this initially sampled time-to-event.

These approaches can be broadly categorised into specific modelling strategies (Barton et al., 2004; Degeling et al., 2019; Degeling et al., 2022).

1. Strategy 1—Event-Specific Distributions (ESD): it involves sampling times to each event and simulating the first event to occur. It uses event-specific distributions to sample time-to-event for each competing event and then selects the earliest to simulate.
2. Strategy 2—Event-Specific Probabilities and Distributions (ESPD): the event type is sampled first based on specific probabilities, followed by sampling the time-to-event from the corresponding distribution. The resulting model is a mixture of event-specific time-to-event distributions, weighted by their probabilities.
3. Strategy 3—Unimodal and Multimodal Distribution and Regression (UDR & MDR): the time-to-event is sampled first, using either a unimodal or multimodal distribution. It then employs a regression model to determine the specific event that corresponds to the sampled time.
4. Strategy 4–using discrete time cycles with transition probabilities: it operates in discrete time cycles and uses transition probabilities for state changes. This strategy resembles a discrete-time state-transition model (Siebert et al., 2012) more than a traditional DES. While it can be useful in certain contexts, it sacrifices the continuous-time advantages and complex event dependencies that DES is designed to capture.

Previous research has focused on the ESD, ESPD, UDR, and MDR modelling strategies in the context of uncensored individual patient data (Degeling et al., 2019), demonstrating that accuracy depended on the number of competing events, overlap of time-to-event distributions for the competing events, and sample size. While these studies have shown that the ESPD approach performs well and is straightforward to implement for uncensored data (Degeling et al., 2019), there is a methodological gap when it comes to censored data, which is a common challenge in long-term studies and real-world settings. The impact of data censoring on model accuracy has been examined for the ESD and UDR approaches (Degeling et al., 2022), but no framework currently exists for implementing the ESPD approach in the presence of censoring.

The primary objective of this study is to adapt the ESPD approach for handling censored data, which will offer several advantages. Firstly, the ESPD approach has proven to be effective and straightforward for uncensored data (Degeling et al., 2019), yet its application is limited by the lack of a framework to handle censored data. Given the commonality of censored data in long-term and real-world studies, our study could significantly expand the method’s applicability. Secondly, while existing methods like ESD and UDR have frameworks to deal with censored data, they do not offer the same advantages as the ESPD in terms of ease of implementation and effective uncertainty estimation around time-to-event parameters. Addressing this limitation involves tackling technical challenges, one of which is the current absence of a well-defined likelihood function tailored for the ESPD approach in censored data scenarios, a gap that our study aims to fill.

By addressing this methodological gap, we provide a more versatile toolset for analysts in this field. On the practical side, we offer implementations of this adapted ESPD approach in both Bayesian and frequentist methods using R, thereby catering to a wide range of statistical preferences and needs. The paper is structured to provide comprehensive evidence for the tailored ESPD approach. We start by defining the ESPD approach in the methods section, followed by a simulation study for accuracy assessment. To demonstrate its utility in real-world scenarios, a case study is included for illustration. The paper concludes with a general discussion that synthesises our findings and outlines directions for future research.

## 2 Methods

We follow standard notation for survival analyses, where T is the event time or censoring time, a continuous random variable that is distributed according to a particular probability density function $f\left(t\right)$ , with cumulative distribution function 1) $F\left(t\right)$ and survival function 2) $S\left(t\right)$ defined as:

$$F\left(t\right)=P\left(T\leq t\right)=\int_{0}^{t}f\left(x\right)dx$$ (1)

$$S\left(t\right)=P\left(T>t\right)=1-F\left(t\right)=\int_{t}^{\infty }f\left(x\right)dx$$ (2)

Furthermore, let K=1,…,k be the index set for k mutually exclusive independent competing events and let $C_{j}$ be the event indicator which shows whether person i,i=1,…,n experienced event j,j=1,…,k , or not. For notational simplicity we encode our events with the vector c , i.e., if competing event j is experienced, then $c_{j}=1,c_{i,i\neq j}=0.$

Building on 1) and 2), we introduce the ESPD strategy. It offers a two-step procedure for generating events in a competing risk scenario. First, the type of event is selected based on some (unknown) mixture proportions. Second, the time of occurrence for the chosen event is sampled from a corresponding survival distribution (e.g., Weibull, Gompertz, *etc.*). Effectively, this results in times that are a *mixture of* m *distributions*, where m=k aligns naturally with the k mutually exclusive independent competing events described in the survival analysis framework. Additionally, we allow both the mixture proportions and the survival distributions to depend on covariates X , such as age, disease stage, *etc.*, thereby making the final model a multivariable mixture model.

To successfully implement the ESPD strategy in the context of censored data, the next step is to define a robust likelihood function. Unlike other methods like ESD or UDR, where frameworks for handling censored data are already established, the ESPD strategy lacks such a framework. As a result, our study introduces a tailored likelihood function, to allow for more accurate and reliable parameter estimation. This involves parameterising two critical components: the mixture proportions or event risks i) and the time-to-event distributions ii).

The first component involves modeling the type of event i). Specifically, we employ a multinomial distribution with event probabilities $\pi =\left(\pi_{1},\ldots ,\pi_{k}\right)$ , such that $\sum_{j=1}^{k}\pi_{j}=1$ . To allow mixture proportions π to depend on some vector of covariates $X^{\pi }$ , a linear relationship is assumed. The model for mixture proportions is constructed using the softmax function, which takes as input the product of the vector of covariates $X^{\pi }$ , and a vector of coefficients $\beta^{\pi }$ . We model it as follows:

$$\pi =softmax\left(\beta^{\pi }X^{\pi }\right)$$ (3)

The relationship between event probabilities and covariates is expressed by the softmax Eq 3. It serves to map the linear combination of predictor variables to a probability set that always sums up to 1. By ensuring this, the function guarantees a positive probability distribution. These probabilities are subsequently used to estimate the mixture proportions or event risks.

The linear combination of the covariates (known from the data) and their coefficients (to be estimated) creates a score (or logit) for each event, which can be represented as $z=\beta^{\pi }X^{\pi }$ . If we consider two competing risks and two covariates, our score vector can be detailed as $z=\left[z_{1},z_{2}\right]$ , where $z_{1}$ represents the score for the first event (e.g., recurrence) and $z_{2}$ ​ is for the second event (e.g., death).

Further, the transformation using the softmax function for a given score *x*_*i*_​ in a vector *x* can be given by $\sigma \left(x_{i}\right)=\frac{e^{x_{i}}}{\sum_{j=1}^{L}e^{x_{j}}}$ , where, *L* corresponds to the total number of events, and *e* is the base of the natural logarithm.

When the transformation is applied to the score vector z , the resulting probabilities for the two events are $\pi_{1}=\frac{e^{z_{1}}}{e^{z_{1}}+e^{z_{2}}}$ and $\pi_{2}=\frac{e^{z_{2}}}{e^{z_{1}}+e^{z_{2}}}$ . Within this context, $\pi_{1}$ gives the probability of the occurrence of the first event, while $\pi_{2}$ provides the probability for the second event.

For the second component ii), conditioned on the occurrence of a specific event j , a particular survival function $S_{j}\left(t,\theta_{j}\right)$ is used to model the time-to-event distribution 4):

$$P\left(T>t|c_{j}=1\right)=S_{j}\left(t,\theta_{j}\right)=\int_{t}^{\infty }f_{j}\left(s,\theta_{j}\right)ds$$ (4)

Different risks may have different distributions and the parameter vectors for these distributions $\theta_{j},j=1,\ldots ,k$ can incorporate dependence on (potentially risk-specific) covariates as well.

For non-censored data 5), the probability of observing an event of type j ( $c_{j}=1$ ) at time t is:

$$P\left(t|c_{j}=1\right)=\pi_{j}f_{j}\left(t,\theta_{j}\right)$$ (5)

While for censored data 6), since no event is observed $(\sum_{j=0}^{k}c_{j}=0)$ , we know that whichever event got selected, the corresponding *“failure”* occurred after the end of the experiment. In other words, no event has occurred yet:

$$P\left(t\;|\sum_{j=0}^{k}c_{j}=0\right)=\sum_{j=1}^{k}\pi_{j}S_{j}\left(t,\theta_{j}\right)$$ (6)

Ignoring covariates for simplicity, the combined likelihood (for k competing risks) can be written as follows:

$$P\left(t,c\left|\pi ,\theta \right.\right)=\underset{A}{\underbrace{\prod_{j=1}^{k}{\left[\pi_{j}f_{j}\left(t,\theta_{j}\right)\right]}^{c_{j}}}}\times \underset{B}{\underbrace{\left[{\left\{\sum_{j=1}^{k}\pi_{j}S_{j}\left(t,\theta_{j}\right)\right\}}^{1-\sum_{i=1}^{k}c_{i}}\right]}}$$ (7)

Here, term in A applies if an event is observed, i.e., $c_{j}=1$ for some j , while B will become 1 since $1_{\sum_{j=1}^{k}c_{j}=0}=0$ ). If no event is observed, i.e., $\sum_{j=0}^{k}c_{j}=0$ , only B will contribute.

In Eq 7, we presented the combined likelihood for handling an arbitrary number k of competing risks. When covariates are involved in the analysis, they would generally be incorporated into both *π* and *θ*, so that each parameter can be parameterised with covariates.

Considering that many practical applications consider two competing risks, k=2 , we also provide the simplified likelihood function 8) for such case. The incorporation of covariates to the likelihood function 8) is implemented in the accompanying R code for both frequentist and Bayesian analyses.

$$P\left(t,c|\pi ,\theta \right)={\left(\pi_{1}f_{1}\left(t,\theta_{1}\right)\right)}^{c_{1}}\times {\left(\pi_{2}f_{2}\left(t,\theta_{2}\right)\right)}^{c_{2}}\times {\left(\pi_{1}S_{1}\left(t,\theta_{1}\right)+\pi_{2}S_{2}\left(t,\theta_{2}\right)\right)}^{1-c_{1}-c_{2}}$$ (8)

When considering the two competing risks setting based on Eq 8, there would be seven parameters to be estimated. These parameters, encompassing coefficients in our expression for π, as well as associated shapes and scales, are intrinsically linked to the observables *t* (time) and *c* (event type). The latter are observations found in the study dataset, while the parameters are to be estimated from the dataset. Our goal is to determine the best-fit parameters that align with the observed data. This entails solving an optimization problem to obtain the maximum likelihood estimates from the dataset.

## 3 Results

### 3.1 Simulation study

A simulation study was performed to verify the accuracy and performance of the ESPD modelling approach. All files related to the simulation study can be accessed on GitHub: https://github.com/koendegeling/CompetingEvents_ESPD.

The considered hypothetical scenario included k=2 competing risks: recurrence (recur) and death before recurrence (death), and we opted for parameter values that are consistent with practical, real-world data, particularly in the oncology setting. The chosen coefficients were selected to reflect realistic relationships between disease stage and time-to-event risks, providing a credible foundation for our model. Simulated patients had equal probabilities of being diagnosed at disease stage IA, IB, or II, and their age was normally distributed, with mean 60 years and a standard deviation of 5 (normalised to mean 0). The true parameter values used to simulate the population were:

$$\log\left(\frac{\pi_{recur}}{1-\pi_{recur}}\right)=-0.4+0.4\;stageIB+0.8\;stageII$$

$$F_{recur}\left(t|\theta_{recur}\right)=Weibull\left(t,\theta_{recur}^{shape}=\exp\left(0.7\right),\theta_{recur}^{scale}=\exp\left(2-0.2\;stageIB-0.6\;stageII\right)\right)$$

$$F_{death}\left(t|\theta_{death}\right)=Gompertz\left(t,\theta_{death}^{shape}=0.1,\theta_{death}^{rate}=\exp\left(-3.5+0.1\;age\right)\right)$$

Based on these true parameters, a population ( $s_{pop}$ ) of $n_{sim}=1,000,000$ individuals was simulated. Subsequently, the performance of the ESPD approach was assessed for a range of scenarios defined by the proportion of censored observations ( $p_{censored}=0.0,0.1,0.3,0.6$ ) and various sample sizes ( $n_{sample}=50,100,200,500$ ) using the following procedure:

• For all combinations of $p_{censored}$ and $n_{sample}$ :
• For $n_{run}=10,000$ iterations:
• Draw a sample $s_{uncensored}$ from population $s_{pop}$ according to $n_{sample}$

• Censor sample $s_{uncensored}$ according to $p_{censored}$ to obtain sample $s_{censored}$

• Analyse $s_{censored}$ according to the ESPD approach
• Based on the estimated parameters, simulate a new sample $s_{sim}$ of size $n_{sim}$

• Assess the performance by comparing the outcomes of $s_{sim}$ to the population $s_{pop}$

Censoring was performed through an independent process where censoring times were sampled from an exponential distribution defined by a censoring rate. If the sampled censoring time was lower than that of the event, the observation was censored at the censoring time. The censoring rate was increased incrementally until the required proportion of censored observations was achieved.

Further, the performance of the approach was assessed in terms of the probability of recurrence, as well as the mean and distribution of the time-to-recurrence and time-to-death. The performance of the event probability and mean time-to-events was quantified using a range of error measures, for which lower values corresponds to a better performance.

• Error ( E ) or bias: E=sim−pop

• Absolute error ( AE ): $AE=\left|sim-pop\right|$

• Relative error ( RE ): $RE=\frac{sim-pop}{pop}$

• Relative absolute error ( RAE ): $RAE=\frac{\left|sim-pop\right|}{pop}$

In which pop refers to the simulated population of individuals with aforementioned characteristics, which is simulated based on the true parameter values, while sim refers to the simulations based on the parameter values as estimated by the ESPD approach.

Lastly, considering these measures do not consider the variance and spread of the distributions of the time-to-events, we also quantified the performance of these distributions by the Kullback-Leibler divergence ( KLD ), or relative entropy, which is widely used to assess the likeliness of distributions (Kullback and Leibler, 1951):

$$KLD\left(f_{pop}\left(t\right)|f_{sim}\left(t\right)\right)=\int_{0}^{\infty }f_{pop}\left(x\right)\log\left(\frac{f_{pop}\left(x\right)}{f_{sim}\left(x\right)}\right)dx=\int_{0}^{\infty }f_{pop}\left(x\right)\times \left(\log\left(f_{pop}\left(x\right)\right)-\log\left(f_{sim}\left(x\right)\right)\right)dx$$

The KLD is a measure of the distance between probability distributions. In general, the smaller the value of KLD , the closer the simulated distribution is to the true population distribution, and the better the model is at representing the data (Cover, 1999). Thus, a smaller KLD indicates a better fit between the simulated and the true distributions, whereas a larger KLD indicates a worse fit.

Given that none of the performance measures considers second-order uncertainty and given that the frequentist implementation is more computationally efficient in obtaining point-estimates compared to the Bayesian implementation, the former was used in the simulation study. Although the Bayesian implementation is illustrated for the case study, a formal comparison of the two implementations in the simulation study was beyond the scope of this study.

Overall, the ESPD approach performed well. We observed that higher proportions of censoring and lower sample sizes both negatively impacted the accuracy of the approach across all performance measures. All results of the simulation study are available in Table 1.

**TABLE 1:** Simulation study results, based on varying degree of censoring (Prop censor), and different sample size (Size). E: error (bias); AE: absolute error; Prob: probability; Prop: proportion; RE: relative error; RAE: relative absolute error; TTR: time-to-recurrence; TTD: time-to-death.

Prop censor	Size	Prob TTR.E	Prob TTR.AE	Prob TTR.RE	Prob TTR.RAE	Mean TTR.E	Mean TTR.AE	Mean TTR.RE	Mean TTR.RAE	Mean TTD.E	Mean TTD.AE	Mean TTD.RE	Mean TTD.RAE	TTR.KLD	TTD.KLD
**0**	50	0.00 (−0.14; 0.14)	0.06 (0.00; 0.16)	0.00 (−0.28; 0.28)	0.11 (0.00; 0.32)	−0.01 (−1.11; 1.16)	0.46 (0.02; 1.29)	0.00 (−0.22; 0.23)	0.09 (0.00; 0.26)	0.03 (−2.71; 2.86)	1.13 (0.04; 3.19)	0.00 (−0.22; 0.23)	0.09 (0.00; 0.26)	0.07 (0.01; 0.24)	0.07 (0.02; 0.23)
**0**	100	0.00 (−0.10; 0.10)	0.04 (0.00; 0.11)	0.00 (−0.20; 0.19)	0.08 (0.00; 0.23)	−0.01 (−0.79; 0.81)	0.32 (0.01; 0.91)	0.00 (−0.16; 0.16)	0.06 (0.00; 0.18)	0.01 (−1.95; 1.99)	0.79 (0.03; 2.23)	0.00 (−0.16; 0.16)	0.06 (0.00; 0.18)	0.04 (0.01; 0.13)	0.05 (0.02; 0.11)
**0**	200	0.00 (−0.07; 0.07)	0.03 (0.00; 0.08)	0.00 (−0.14; 0.14)	0.06 (0.00; 0.16)	0.00 (−0.55; 0.56)	0.22 (0.01; 0.63)	0.00 (−0.11; 0.11)	0.04 (0.00; 0.13)	0.00 (−1.39; 1.39)	0.57 (0.02; 1.59)	0.00 (−0.11; 0.11)	0.05 (0.00; 0.13)	0.03 (0.01; 0.08)	0.04 (0.02; 0.07)
**0**	500	0.00 (−0.04; 0.04)	0.02 (0.00; 0.05)	0.00 (−0.09; 0.09)	0.04 (0.00; 0.10)	0.00 (−0.34; 0.35)	0.14 (0.01; 0.39)	0.00 (−0.07; 0.07)	0.03 (0.00; 0.08)	0.01 (−0.86; 0.86)	0.35 (0.01; 0.98)	0.00 (−0.07; 0.07)	0.03 (0.00; 0.08)	0.03 (0.01; 0.05)	0.03 (0.02; 0.05)
**0.1**	50	0.00 (−0.15; 0.15)	0.06 (0.00; 0.17)	0.00 (−0.30; 0.29)	0.12 (0.00; 0.34)	0.01 (−1.17; 1.35)	0.51 (0.02; 1.46)	0.00 (−0.24; 0.27)	0.10 (0.00; 0.29)	0.06 (−2.91; 3.10)	1.22 (0.05; 3.48)	0.00 (−0.23; 0.25)	0.10 (0.00; 0.28)	0.08 (0.01; 0.29)	0.08 (0.02; 0.29)
**0.1**	100	0.00 (−0.10; 0.10)	0.04 (0.00; 0.12)	0.00 (−0.21; 0.20)	0.08 (0.00; 0.24)	0.00 (−0.83; 0.86)	0.34 (0.01; 0.97)	0.00 (−0.17; 0.17)	0.07 (0.00; 0.20)	0.02 (−2.06; 2.08)	0.85 (0.03; 2.40)	0.00 (−0.17; 0.17)	0.07 (0.00; 0.19)	0.05 (0.01; 0.14)	0.05 (0.02; 0.14)
**0.1**	200	0.00 (−0.07; 0.07)	0.03 (0.00; 0.08)	0.00 (−0.14; 0.14)	0.06 (0.00; 0.16)	0.00 (−0.58; 0.59)	0.24 (0.01; 0.68)	0.00 (−0.12; 0.12)	0.05 (0.00; 0.14)	0.01 (−1.48; 1.47)	0.60 (0.02; 1.70)	0.00 (−0.12; 0.12)	0.05 (0.00; 0.14)	0.03 (0.01; 0.08)	0.04 (0.02; 0.08)
**0.1**	500	0.00 (−0.05; 0.04)	0.02 (0.00; 0.05)	0.00 (−0.09; 0.09)	0.04 (0.00; 0.10)	0.00 (−0.36; 0.37)	0.15 (0.01; 0.41)	0.00 (−0.07; 0.07)	0.03 (0.00; 0.08)	0.02 (−0.90; 0.94)	0.37 (0.01; 1.04)	0.00 (−0.07; 0.08)	0.03 (0.00; 0.08)	0.03 (0.01; 0.05)	0.03 (0.02; 0.05)
**0.3**	50	0.00 (−0.16; 0.18)	0.07 (0.00; 0.20)	0.00 (−0.33; 0.36)	0.14 (0.01; 0.40)	0.38 (−1.35; 2.99)	0.94 (0.03; 2.99)	0.08 (−0.27; 0.60)	0.19 (0.01; 0.60)	0.08 (−3.98; 4.11)	1.59 (0.05; 4.78)	0.01 (−0.32; 0.33)	0.13 (0.00; 0.38)	0.12 (0.01; 0.45)	0.15 (0.03; 0.76)
**0.3**	100	0.00 (−0.12; 0.12)	0.05 (0.00; 0.13)	0.00 (−0.23; 0.23)	0.09 (0.00; 0.27)	0.02 (−0.95; 1.17)	0.42 (0.02; 1.23)	0.00 (−0.19; 0.23)	0.08 (0.00; 0.25)	0.06 (−2.43; 2.67)	1.03 (0.04; 2.94)	0.00 (−0.20; 0.22)	0.08 (0.00; 0.24)	0.06 (0.01; 0.19)	0.07 (0.02; 0.24)
**0.3**	200	0.00 (−0.08; 0.08)	0.03 (0.00; 0.09)	−0.01 (−0.16; 0.15)	0.06 (0.00; 0.18)	−0.01 (−0.68; 0.71)	0.28 (0.01; 0.78)	0.00 (−0.14; 0.14)	0.06 (0.00; 0.16)	0.11 (−1.58; 1.79)	0.69 (0.03; 1.95)	0.01 (−0.13; 0.14)	0.06 (0.00; 0.16)	0.04 (0.01; 0.10)	0.05 (0.02; 0.10)
**0.3**	500	0.00 (−0.05; 0.05)	0.02 (0.00; 0.06)	0.00 (−0.10; 0.10)	0.04 (0.00; 0.12)	0.00 (−0.42; 0.43)	0.17 (0.01; 0.49)	0.00 (−0.08; 0.09)	0.03 (0.00; 0.10)	0.07 (−0.99; 1.13)	0.43 (0.02; 1.21)	0.01 (−0.08; 0.09)	0.03 (0.00; 0.10)	0.03 (0.01; 0.06)	0.04 (0.02; 0.06)
**0.6**	50	0.02 (−0.22; 0.33)	0.11 (0.01; 0.33)	0.05 (−0.44; 0.66)	0.23 (0.01; 0.66)	3050.09 (−1.76; 24.99)	3050.68 (0.05; 24.99)	612.99 (−0.35; 5.02)	613.10 (0.01; 5.02)	−0.15 (−7.55; 9.81)	3.11 (0.11; 10.21)	−0.01 (−0.61; 0.79)	0.25 (0.01; 0.82)	0.26 (0.02; 1.00)	0.49 (0.03; 2.97)
**0.6**	100	0.00 (−0.17; 0.24)	0.08 (0.00; 0.24)	−0.01 (−0.34; 0.48)	0.16 (0.01; 0.49)	1.15 (−1.36; 5.45)	1.70 (0.03; 5.45)	0.23 (−0.27; 1.09)	0.34 (0.01; 1.09)	0.44 (−5.56; 6.83)	2.21 (0.08; 7.47)	0.04 (−0.45; 0.55)	0.18 (0.01; 0.60)	0.14 (0.01; 0.45)	0.23 (0.03; 1.52)
**0.6**	200	−0.02 (−0.13; 0.12)	0.05 (0.00; 0.14)	−0.03 (−0.25; 0.24)	0.10 (0.00; 0.29)	0.18 (−1.00; 2.42)	0.61 (0.02; 2.42)	0.04 (−0.20; 0.49)	0.12 (0.00; 0.49)	0.73 (−2.75; 5.34)	1.57 (0.06; 5.51)	0.06 (−0.22; 0.43)	0.13 (0.00; 0.44)	0.07 (0.01; 0.25)	0.10 (0.02; 0.41)
**0.6**	500	−0.01 (−0.08; 0.06)	0.03 (0.00; 0.08)	−0.02 (−0.16; 0.13)	0.06 (0.00; 0.17)	0.02 (−0.64; 0.78)	0.29 (0.01; 0.82)	0.00 (−0.13; 0.16)	0.06 (0.00; 0.17)	0.40 (−1.44; 2.53)	0.85 (0.03; 2.55)	0.03 (−0.12; 0.20)	0.07 (0.00; 0.21)	0.04 (0.01; 0.09)	0.05 (0.02; 0.14)

Regarding the event probability, on average, there was no error in the E or RE up to 30% censoring. For 60% censoring, the RE ranged from −0.02 (95% confidence interval: 0.16; 0.13) for sample size 500, to 0.05 (−0.44; 0.66) for size 50. The average RAE ranged from 0.04 (0.00; 0.10) for multiple scenarios, to 0.23 (0.01; 0.66) for 60% censoring and size 50.

For the mean time-to-event, similar as for the event probability, on average there was basically no error in the E or RE up to 30% censoring. For 60% censoring, unrealistic results were obtained for sample size 50. Other than that, for 60% censoring, the average RE ranged from 0.00 (−0.13; 0.16) for time-to-recurrence and size 500, to 0.23 (−0.27; 1.09) for time-to-recurrence and size 100. Excluding the scenario of 60% censoring and size 50, the average RAE ranged from 0.03 (0.00; 0.08) for multiple scenarios, to 0.34 (0.01; 1.09) for time-to-recurrence under 60% censoring and for size 100.

In terms of the KLD , similar trends were observed. The KLD ranged from 0.03 (0.01; 0.05) for multiple scenarios, to 0.49 (0.03; 2.97) for time-to-death for the scenario of 60% censoring and size 50. As the KLD values were relatively close to 0 for most scenarios, this suggested that the ESPD model performed well in approximating the true population distribution of time-to-events. However, for the scenario with 60% censoring and sample size 50, the KLD value for time-to-death was relatively larger than 0.1 and higher compared to other scenarios, thus suggesting that the model did not fit the data as well, which is a similar pattern observed with alternative modelling strategies.

### 3.2 Case study

The overall aim of the case study is to provide users with an understanding of the steps involved in implementing the ESPD strategy in a simple example in R. This is provided for both frequentist and Bayesian frameworks and considering Weibull distribution only, for simplicity. In this section, we describe key steps towards the ESPD Weibull implementation as an illustration, and advice the reader to refer to the publicly available scripts for a full description and detailed step-by-step implementation of the strategy. Finally, so that users can fully apply the ESPD approach, we also provide a custom R function that allows for fitting various distributions, including other than Weibull, together with visual fits and Akaike Information Criterion (AIC) scores. These files are also accessible on the listed GitHub repository.

We use a publicly available dataset *melanoma*, available from the *boot* package in R (Hinkley and Anthony, 1997; Ripley and Angelo, 2022). The *melanoma* dataset was originally analysed by Andersen et al. (1993) and consists of measurements made on patients with malignant melanoma, which all had their tumour removed by surgery in Denmark from 1962 to 1977 (Andersen et al., 1993). Several covariates are available, as summarised in Table 2. In terms of outcomes, we consider the following patient status: deceased, disease recurrence, and alive without disease recurrence (i.e., censored).

**TABLE 2:** Summary of the data used from the publicly available melanoma dataset.

Variable	N = 205 [n (%); median (IQR)]
Demographics	
Sex (male)	79 (39%)
Diagnosis age (years)	54 (42, 65)
Tumour thickness (mm)	1.94 (0.97, 3.56)
Ulcerated tumour	90 (44%)
Outcomes	
Time to last status assessment (years)	5.5 (4.2, 8.3)
Patient status	
Deceased	57 (28%)
Recurred	134 (65%)
Censored	14 (6.8%)

In estimating the probability of recurrence and distributions of the time-to-recurrence and time-to-death, we assume that.

• The mixture proportions are modelled based on covariates age, sex, ulceration status, and tumour thickness,
• Weibull distributions are appropriate for the time-to-event distributions, assuming a single shape parameter across all groups, where the scale parameter is modelled based on the same covariates as the mixture proportions.

#### 3.2.1 Frequentist implementation

The first step is to define the log-likelihood function in R. The *step_by_step_frequentist* notebook provides a thorough step-by-step implementation of the log-likelihood from its simplest version to incorporating censoring, followed by adding covariates, and finally to considering two competing risks. We highly recommend users who are unfamiliar with these concepts to go through the R code and different steps in the notebook.

Box 1 defines the function in its complete form, which returns the log-likelihood for a set of parameters given the data.

BOX 1 **Definition of the log-likelihood function, incorporating the competing events and covariates.**

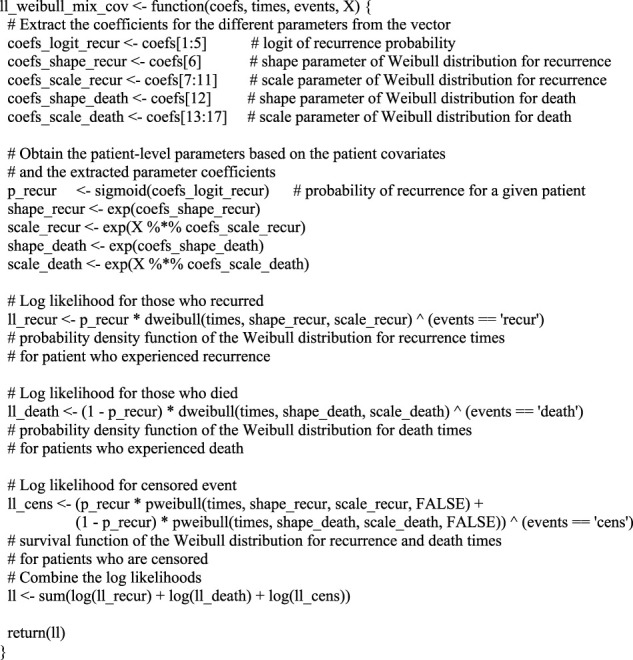

Here, vector t contains the event or censoring times, vector e contains the event data (possible values: recur for disease recurrence, death for deceased patients, or cens for censored patients), and X is the covariance matrix. Furthermore, coefs represents the vector of coefficients that are to be estimated, which need to be defined through a single vector for most optimisation functions. Therefore, the first step in the function is to extract the coefficients for the different parameters from the coefs vector. For each parameter that is modelled based on the 4 covariates, there are 5 coefficients: 1 for the intercept and one for each covariate. For the shape parameters that are not modelled based on covariates, there simply is one coefficient. Subsequently, the coefficients are transformed into the parameters for the mixture distribution. Transformations are required because the mixture proportions π are modelled as a logistic regression model and the resulting log-odds need to be transformed to probabilities. Similarly, the shape and scale parameters of the Weibull distributions need to be non-negative and are, therefore, typically modelled using coefficients that are log-transformed. That way, the coefficients can have any negative or positive value in the optimization process, whilst the corresponding parameters will be non-negative. In R, the %*% operator is used for matrix multiplications. This is used in the code to apply covariate matrix X to the coefficients, resulting in vectors of patient-specific parameter values, such as p_recur , shape_recur , *etc.* For readability of the code, the log-likelihood is obtained in 3 separate steps, one for each of the possible events.

The next step is to apply the ll_weibull_mix_cov function to the data to find the optimal coefficients by maximizing the likelihood function. For this we use the maxLik function of the maxLik package (Henningsen et al., 2010), which was developed with this exact objective in mind, and which conveniently returns the variance-covariance matrix together with the coefficient estimates. In this function, we need to specify the log-likelihood function, start values for the coefficients, and any arguments that need to be passed on to the function, which are *t, e,* and *X* in this case. Because we optimize the function defined by coefficients and not the parameters on real scale, we can simply specify a zero as the starting value for each parameter. Once the optimization is performed, point estimates for the coefficient values can be extracted from the optimization object. This process is illustrated in Box 2.

BOX 2 **Performing the maximisation of the likelihood function and extracting the results.**

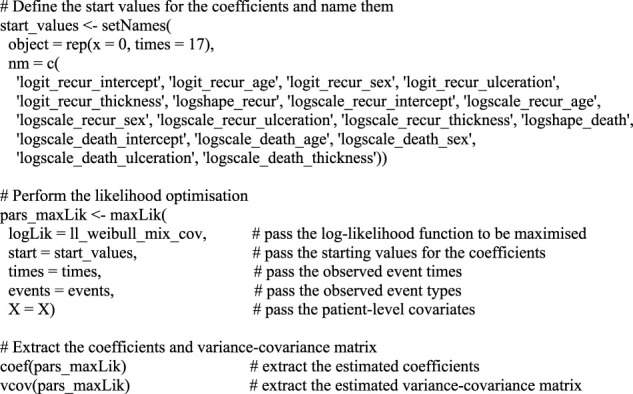

Although this step-by-step implementation in R is relatively straightforward, a general function that can be used to apply the ESPD approach for modelling two competing events has been made available with the tutorial on GitHub. The function is available in the script ESPD_frequentist.R, providing all the functionality one may require, such as allowing for different parametric families of distributions for individual risks. Further information is available in the corresponding script.

#### 3.2.2 Bayesian implementation

The Bayesian implementation is fully detailed in the notebook *case_study_bayesian.Rmd*, together with the Stan model *weibull_mix_cov.stan* on GitHub. Stan is a probabilistic programming language for specifying statistical models, providing full Bayesian inference, approximate Bayesian inference and penalised maximum likelihood estimation with optimisation (Team, 2023). In R, Stan can be called through various libraries and in this implementation, we use *CmdStanR* (Češnovar, 2022), which does not interface directly with C++ and is thus user friendly for beginners. In Stan, a typical simulation is a two-step process, by which we first fit the model on existing data to obtain posterior estimates of all parameters, and then sample from the resulting distribution to obtain a synthetic dataset.

The Bayesian implementation inherently captures parameter uncertainty in a principled manner through posterior distributions. These distributions can be used directly to inform parameter values in a probabilistic analysis of the simulation model (Briggs et al., 2012).

#### 3.2.3 Case study results

In this section, we highlight the application and interpretation of Bayesian implementation outcomes. Though the conclusions are applicable to the frequentist case, we believe this example presents an educational opportunity for readers to compare optimisation (Box 2) results in R. This demonstration’s objective is less about unearthing groundbreaking findings and more about illuminating the nuances of a practical implementation.

**FIGURE 1:**
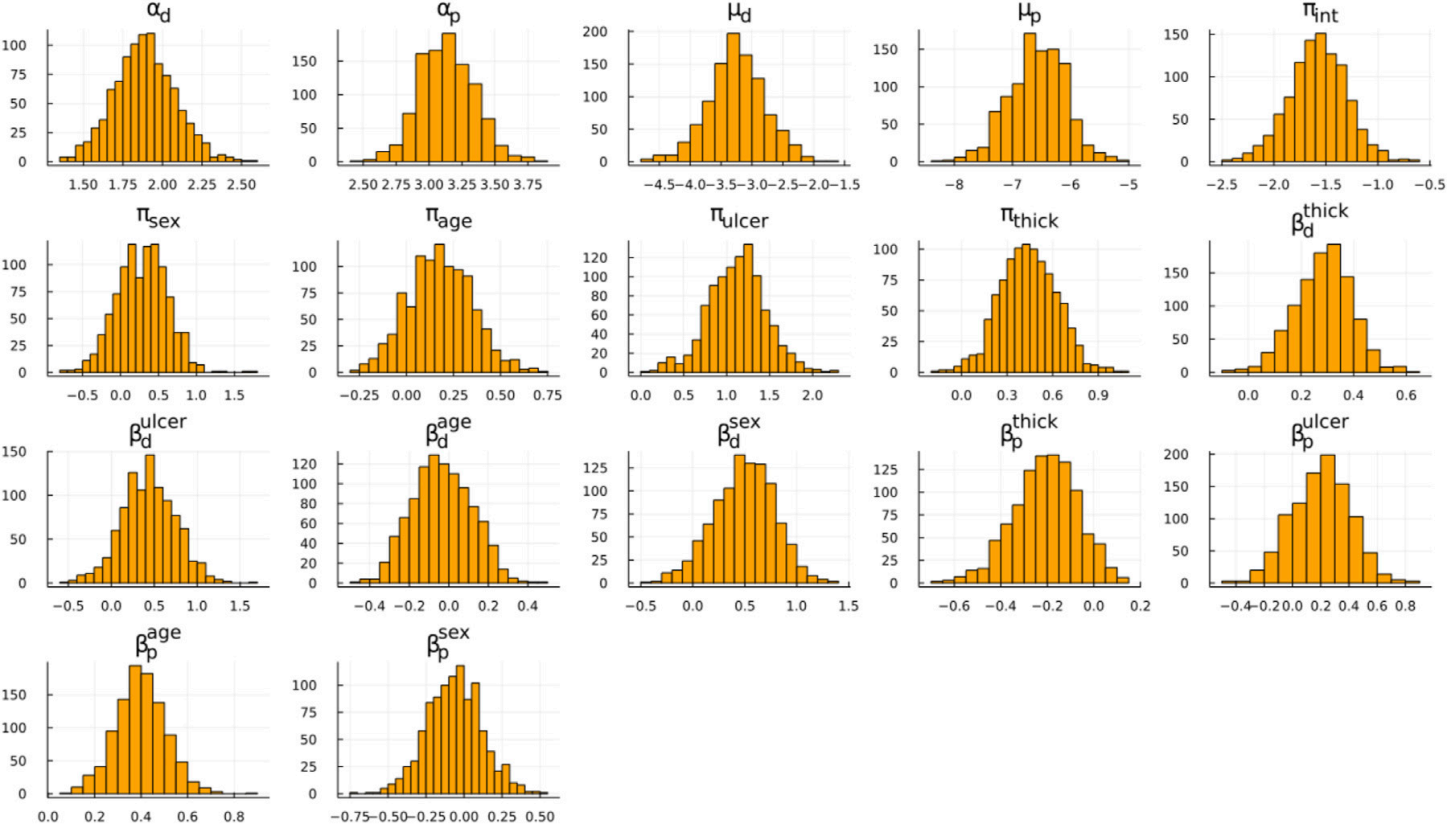
Posterior distribution samples for the ESPD model. Weibull parameters: α, μ; Mixing proportion: π; Adjustment coefficients: β; Subscripts: *p* (recurrence), *d* (death).

Our discussion focuses on Figure 1; Table 3. In Figure 1, the posterior distribution of various model parameters is displayed. On the x-axis, we see the parameter values, and the y-axis portrays their density. These parameters are part of the Bayesian ESPD model for competing risks, which uses Weibull distributions. Each event, be it recurrence or death, has its parameters estimated individually. In these plots, the parameters alpha (α) and mu (μ) are prominent, serving as the fundamental shape and scale components of the Weibull distribution. In contrast, beta (β) encompasses the regression coefficients tied to the model’s covariates, and the pi (π) parameter defines coefficients for the mixture proportions derived from the covariates.

**TABLE 3:** Mean posterior probabilities for recurrence event for each censored individual.

Age	Sex	Thickness	Ulcer	Time to last status assessment (years)	Probability of recurrence
76	Male	6.76	Present	0.03	0.35
56	Male	0.65	Absent	0.08	0.83
71	Female	2.90	Absent	0.27	0.80
60	Female	3.22	Present	0.64	0.59
64	Female	0.16	Present	0.97	0.68
72	Male	12.56	Present	1.35	0.29
86	Female	8.54	Present	2.26	0.44
64	Male	1.29	Absent	3.91	0.87
76	Female	1.29	Present	4.18	0.70
71	Male	4.84	Present	5.10	0.84
66	Female	0.65	Absent	5.71	0.88
49	Male	1.62	Absent	8.64	0.97
49	Male	6.12	Absent	8.72	0.99
54	Female	1.45	Absent	9.47	0.89

By analysing the coefficients for both risks side-by-side, we aimed to derive an intuitive understanding of their implications. The relationship between these variables and event types can be observed through their respective coefficient values. Specifically, the magnitude and direction of their coefficient values in the model offer insights into their relationships with the event types. A higher coefficient value for a variable suggests a stronger association with the outcome.

A particularly relevant observation from Figure 1 is the mixing proportion intercept (π_int_) with its mean value hovering around −1.5, indicating a higher likelihood of recurrence as opposed to death. Figure 1 also underscores that ulceration and thickness are pivotal factors influencing the outcomes, which can be seen based on the β coefficients. These coefficients depict how changes in ulceration and thickness are associated with changes in the hazard of the events. Furthermore, by examining the π coefficients for these covariates, we can glean insights into their influence on the likelihood of one event type over another, such as recurrence *versus* death.

Examining the Weibull parameters (α and μ), we conclude that the mean death time is shorter than the recurrence time. For a complete view of the distribution, we suggest readers to simulate times for death and recurrence based on mean Weibull parameters with zero covariate effects and plotting a histogram. This can be accomplished using the rweibull_cov function from GitHub. Finally, the likelihood of recurrence increases with the observation period length.

In Table 3, we present the probabilities of recurrence for censored individuals from the melanoma dataset, as sampled from the Bayesian posterior distribution. Recurrence is frequently the more probable outcome. The primary covariates influencing both the event type and its timing are ulceration and thickness, reflecting findings from Figure 1. Patients with a tumour thickness significantly above the mean exhibit a heightened risk of death before recurrence, indicating that thickness may be an indication of melanoma severity.

## 4 Discussion

Competing risks data are common in medical research that aims to investigate an outcome of interest and, hence, decision-analytic models of healthcare pathways commonly include multiple competing events. For example, in oncology, recurrence is a competing risk to death prior to recurrence, which is typically modelled based on background mortality. Here, we addressed a methodological gap by defining and illustrating a modelling approach for implementing the ‘event first, time second’ strategy for modelling competing events in DES when the parameters are to be estimated based on censored data. The resulting ESPD modelling approach was mathematically defined for any number of competing risks in Eqs 7, 8, and implementations in both the frequentist and Bayesian framework were provided for two competing risks, including when considering covariates. Finally, the approach was evaluated in a simulation study and illustrated in a case study for which the corresponding R code has been made available with this manuscript.

The results of the simulation study indicate that the frequentist implementation of the ESPD approach performs well under various degrees of censoring and sample sizes. However, its accuracy diminishes with decreasing sample sizes and increasing levels of censoring (Table 1). These results are consistent with past studies on implementing competing events in DES with uncensored and censored data using alternative strategies (Degeling et al., 2019; Degeling et al., 2022). Our findings reiterate the importance for modellers to recognise that datasets characterised by high censoring levels and small sample sizes might render the ESPD approach less reliable for simulations, which also holds for other methods previously investigated (Degeling et al., 2019). Although a formal comparison of the frequentist and Bayesian implementations was beyond the scope of the simulation study, the case study demonstrated that both implementations yielded comparable and realistic results. Further research may compare the frequentist and Bayesian implementations more systematically to identify whether either may be preferable in certain scenarios. Significantly, our study introduces an additional method to the existing techniques for addressing censoring, filling the gap where no method was previously delineated for such censoring.

Further research is also warranted to quantitatively compare the performance of the ESPD to previously defined modelling approaches for implementing competing events in DES based on censored data (Degeling et al., 2022), in line with previous work focused on uncensored data (Degeling et al., 2019). This would also inform selection between the different modelling approaches. Such guidance is already available for the ESD, ESPD, UDR and MDR approaches for scenarios in which they are informed by uncensored data, as well as for the ESD and UDR approaches when informed by censored data. Based on the previous work for uncensored data and the results of our simulation study, we expect that the ESPD approach will have good accuracy and be relatively straightforward to implement and interpret when used for censored data compared to the other approaches, but this is to be confirmed in a comparative simulation study. In this context, it is important to note that the interpretation of likelihood-based measures, such as the AIC may be different between the approaches. For the ESD approach, the likelihood only considers the time-to-event for each event independently and not the type-of-event, whereas the likelihood in the UDR approach considers the likelihood of the time-to-event and event-type separately, and the ESPD considers the time-to-event and event-type of all events jointly. Regardless, despite high-level guidance on the selection of different approaches being useful, validation of the results will remain pertinent in the modelling process.

By demonstrating the implementation of the ESPD approach using both the frequentist and Bayesian frameworks in R and Stan, we enable novice and more advanced R users to leverage this modelling strategy. The frequentist implementation allows for a fast and relatively easy retrieval of the point estimates and the variance-covariance matrix of the coefficient, especially with the provided general functions that facilitates incorporation of covariates and different distribution types. Whilst more challenging to implement, some may argue that the Bayesian version provides a more natural and principled way of combining useful prior information into the estimate, which may be more accurate than a frequentist estimate, if such information is available. Furthermore, some consider the interpretation of a Bayesian result more straightforward, as it provides a framework about the unknown parameter conditional on the observed data, rather than about the observed data conditional on the unknown parameter. By providing both implementations, we provide modellers with the freedom to use the framework they prefer.

The ESPD approach was developed for modelling competing events in DES. However, the event-specific probabilities may also be considered as cumulative event incidences in an epidemiological context. The cumulative incidence of competing risks has generally been modelled using cause-specific hazard models and sub-distribution hazard models (Fine and Gray, 1999; Pintilie, 2006; Lau et al., 2009; Austin and Fine, 2017). The ESPD approach may provide an interesting alternative to these models, where the cumulative event incidences can be modelled directly as the mixture proportions. This could be utilized for estimating probabilities of treatment sequences from real-world data where typically a substantial proportion of patients is still on treatment, i.e., censored for the competing events of switching to a subsequent treatment line and death without further treatment. This is relevant for disease areas where patients typically receive multiple lines of therapy, such as oncology.

In summary, our study has filled a methodological gap by providing a tutorial and framework for modelling competing events in discrete event simulations with censored data. The ESPD approach, which samples the event first and time-to-event second, was found to be accurate and produced realistic results in both simulation and case studies. The ESPD approach has been implemented in both a frequentist and Bayesian framework using R, making it easily accessible for others to use and expand upon in future research. Not only is the ESPD strategy applicable for modelling competing events in DES, but it also has potential to be used in other contexts to estimate cumulative event incidences. Future studies should perform and report on cross-validation of the ESPD approach compared to the other strategies, which will ultimately ensure individual patient data are appropriately modelled.

## Data Availability

The original contributions presented in the study are included in the article/Supplementary material, further inquiries can be directed to the corresponding author.
